# Design of Simplified Maximum-Likelihood Receivers for Multiuser CPM Systems

**DOI:** 10.1155/2014/174294

**Published:** 2014-01-27

**Authors:** Li Bing, Baoming Bai

**Affiliations:** State Key Laboratory of ISN, Xidian University, Xi'an 710071, China

## Abstract

A class of simplified maximum-likelihood receivers designed for continuous phase modulation based multiuser systems is proposed. The presented receiver is built upon a front end employing mismatched filters and a maximum-likelihood detector defined in a low-dimensional signal space. The performance of the proposed receivers is analyzed and compared to some existing receivers. Some schemes are designed to implement the proposed receivers and to reveal the roles of different system parameters. Analysis and numerical results show that the proposed receivers can approach the optimum multiuser receivers with significantly (even exponentially in some cases) reduced complexity and marginal performance degradation.

## 1. Introduction

As an efficient constant envelope modulation scheme, continuous phase modulation (CPM) has gained extensive attention since it was developed in 1980s [1] and has been proven to have outstanding performance in single user [2–5] and multiuser systems [6–9]. Compared with conventional multiuser systems based on linear modulations [10, 11], an extraordinary property of CPM is the constant envelope and thus the ability to overcome the nonlinear distortion introduced by a class-C amplifier. This property makes CPM an attractive scheme in modern wireless systems.

While increasing the efficiency, the optimum multiuser receiver consisting of a front-end followed by a detector has a considerable complexity. Generally, the optimum receiver such as maximum-likelihood (ML) multiuser receiver suffers from the exponentially increased complexity (with the number of users) and is considered too complicated to be practical. To reduce the complexity, some suboptimum receivers were proposed [7]. The main strategy is simplifying the detector at the expense of acceptable performance loss in terms of bit error rate (BER). With properly designed parameters such as increasing the frequency separation, even a suboptimal receiver can successfully narrow the gap between suboptimum and optimum receivers [7]. As a matter of fact, the design of front-end for CPM-based multiuser systems gains less attention. The front-end is generally interpreted as an independent module generating sufficient statistics. The most existing designs focus on the techniques of oversampling [1] and generalized Laurent decomposition (LD) (see [12] and references therein). Since they are not particularly suited for multiuser systems, the complexity of such a front-end is linear to the number of users, which probably finally exceeds the capacity of receivers.

In this paper, the joint design of simplified ML receivers is considered. A simplified front-end is designed with purposely incorporated performance loss. The detector followed is a ML detector defined in a low-dimensional signal space and thus complexity reduction can be obtained instantly. The parameters of the receiver are then optimized such that the performance loss is minimized for a given complexity reduction. The main tools employed are the principal component analysis [13] and mismatched filters [14, 15]. The performance of the proposed receiver is measured by the average energy loss, the minimum achievable Euclidean distance, and the total complexity reduction.

This paper is organized as follows. The system model is presented in Section 2, Section 3 presents the simplified receivers and compares its complexity with existing receivers, Section 4 gives the numerical results, and Section 5 concludes the paper.

## 2. System Model

**Figure 1:**
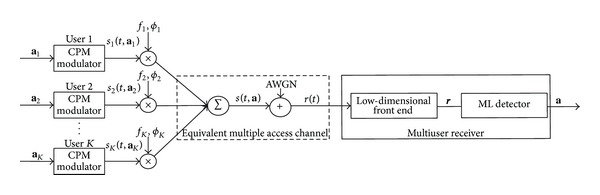
System model.

A multiple-access-channel (MAC) type multiuser CPM system consisting of *K* users is shown in Figure 1. It is assumed that all users employ an identical CPM scheme, where the modulation parameters such as the transmitted energy *E*, modulation level *M*, modulation index *h*, and phase response *q*(*t*) are the same. The *k*th (1 ≤ *k* ≤ *K*) user maps its information sequence **a**_*k*_ = {*a*_*k*1_, *a*_*k*2_,…, *a*_*kN*_} independently to the transmitted signal, whose equivalent complex baseband signal reads [1]

(1) $$\begin{array}{c} s_{k}\left(t,a_{k}\right)=\sqrt{\frac{2E}{T}}\exp j\left\{\varphi \left(t,a_{k}\right)+2\pi f_{k}t+\phi_{k}\right\}, \end{array}$$

where *T*, *E*, and *q*(*t*) are the symbol duration, signal energy and phase response, respectively. The information-bearing phase is

(2) $$\begin{array}{c} \varphi \left(t,a_{k}\right)=\left\{2\pi h\underset{i}{\sum }a_{ki}q\left(t-iT\right)\right\}, \end{array}$$

where *h* = *k*/*p* is the modulation index and the transmitted symbol *a*_*ki*_ ∈ {±1, ±3,…, ±(*M* − 1)}. The phase response function *q*(*t*) is defined as

(3) $$\begin{array}{c} q\left(t\right)=\overset{t}{\underset{-\infty }{\int }}g\left(\tau \right)d\tau , \end{array}$$

where *g*(*τ*) is the frequency response assumed to be causal and of duration *L* symbols. The phase response has the following property:

(4) $$\begin{array}{c} q\left(t\right)=\{\begin{array}{cc} \frac{1}{2} & t\geq LT\\ 0 & t<0. \end{array} \end{array}$$

The frequency separation *f*_*k*_ and phase separation *ϕ*_*k*_ are

(5) $$\begin{array}{c} f_{k}=\Delta f\left(k-\frac{K+1}{2}\right),\\ \phi_{k}=\Delta \phi \left(k-\frac{K+1}{2}\right), \end{array}$$

where Δ*f* and Δ*ϕ* are frequency spacing and phase spacing [6], respectively. According to the model, the superimposed signal (or the equivalent MAC signal) reads

(6) $$\begin{array}{c} s\left(t,a\right)=\overset{k=K}{\underset{k=1}{\sum }}s_{k}\left(t,a_{k}\right). \end{array}$$

## 3. Design of Simplified Receivers

As it was mentioned above, the basic idea is to employ a receiver defined in a low-dimensional signal space, which is optimized such that the minimum achievable distance is maximized. The low-dimensional receiver here is a generalized form of the one presented in [14, 15], where the receiver is built upon a shortened frequency response scheme. The details are presented below.

### 3.1. Principles of the Proposed Receivers

The transmitted signal of the *k*th user is defined as *s*_*k*_(*t*, **a**_*k*_), which reads

(7) $$\begin{array}{c} s_{k}\left(t,a_{k}\right)=\sqrt{\frac{2E_{k}}{T}}\exp\left\{\varphi_{k}\left(t-\tau ,a_{k}\right)+2\pi f_{k}\left(t-\tau \right)+\phi_{k}\right\} \end{array}$$

and the corresponding alternative in received signal space is written as

(8) $$\begin{array}{c} s_{Rk}\left(t,a_{k}\right)=\sqrt{\frac{2E_{k}}{T}}\exp\left\{\psi_{k}\left(t,a_{k}\right)+2\pi f_{k}t+\phi_{k}+\theta \right\}, \end{array}$$

where *ψ*(*t*, **a**_*k*_) is based on a shorter frequency response *g*_*R*_(*t*) whose duration is *L*_*R*_ (*L*_*R*_ ≤ *L*). The quantities *θ* and correct timing *τ* are incorporated to achieve the best fitness between the transmitted and received phase trajectories [14, 15]. The alternative superimposed signal is *s*_*R*_(*t*, **a**) = ∑_*k*=1_^*k*=*K*^ *s*_*Rk*_(*t*, **a**_*k*_), and thus the received signal space is {*s*_*R*_(*t*, **a**)}. Obviously, the size of the signal set is reduced from *M* ^*KL*^ down to *M* ^*KL*_*R*_^ and results in a *M* ^*K*(*L*−*L*_*R*_)^-fold complexity reduction. As a special case when *L*_*R*_ = *L*, there is no complexity reduction in the detector. However, it is still possible to simplify the front-end as we will see below.

Unfortunately, the number of filters required is *M* ^*KL*_*R*_^ which still might be unacceptable. Therefore, the principal components analysis is introduced into the front-end to further reduce the complexity. As we shall see later, the number of the filters can now be reduced significantly.

The proposed scheme is summarized below:

1. find the *θ*, the optimum *q*_*R*_(*t*), and the correcting timing *τ*;
2. calculate the orthogonal basis of {*s*_*R*_(*t*, **a**)} and those with nonzero eigenvalues are considered effective (this is equivalent to determine the effective dimensions and effective basis), the number of which is designated as *N*_*E*_;
3. the front-end, that is, a bank of matched filters, is built upon the effective basis **β**(*t*) = {*β*_1_(*t*),…, *β*_*N*_*E*__(*t*)};
4. the sufficient statistics **r**(w.r.t.  {*s*_*R*_(*t*, **a**)}) are generated and sent to the detector followed;
5. the ML detector delivers the detected $\hat{a}$ of **a**.

For more details of the calculations and parameter optimization, see [14, 15]. It should be pointed out that the resulting receiver is rather versatile. It can be optimum (*L* = *L*_*R*_) or suboptimum (*L* < *L*_*R*_) depending on the signal space {*s*_*R*_(*t*, **a**)} being considered. In the rest of this paper, we focus on the ML detector (w.r.t. {*s*_*R*_(*t*, **a**)}) to evaluate the asymptotic performance.

### 3.2. Performance Measurements

Three measurements are considered in this paper: the minimum achievable distance, the average energy loss, and the number of effective dimensions. The minimum distance is principally the same as in single user systems [14, 15], which reads

(9) $$\begin{array}{c} d^{2}=\underset{a\neq b}{\min}\frac{1}{2E_{b}}{\left[\frac{{\left|s\left(t,a\right)-s_{R}\left(t,b\right)\right|}^{2}-{\left|s\left(t,a\right)-s_{R}\left(t,a\right)\right|}^{2}}{\left|s_{R}\left(t,a\right)-s_{R}\left(t,b\right)\right|}\right]}^{2}, \end{array}$$

where *E*_*b*_ is the average transmitted energy per information bit. It is noticed that *d* ^2^ is positive by definition but is not additive. Therefore, no efficient method but exhaustive search is employed to find this quantity in most cases.

The average energy loss is defined as

(10) $$\begin{array}{c} ɛ=\frac{1}{M^{KL}}\left[\underset{a}{\sum }1-\frac{{\left|\langle s\left(t,a\right),\beta \left(t\right)\rangle \right|}^{2}}{{\left|s\left(t,a\right)\right|}^{2}}\right], \end{array}$$

where 〈, 〉 designates the inner product operation. The quantity *ɛ* is essentially the energy loss projecting *s*(*t*, **a**) to the low-dimensional signal space, averaged over the transmitted signal set.

The number of effective dimensions implies the number of complex filters required by the front-end. This quantity is usually defined as the number of nonzero eigenvalues [13]. It is now redefined as the number of eigenvalues greater than 10^−4^ for practical purpose. It is obvious that such an operation does not undermine the accuracy of the front-end. On the other hand, LD-based receivers also exploited a similar idea but the real-valued filters required usually are defined over durations of several symbol intervals [12, 16].

### 3.3. Complexity

It should be evident from the discussion above that the proposed receiver can reduce the complexity exponentially from *M* ^*KL*^ down to *M* ^*KL*_*R*_^. This results in two kinds of complexity reduction: the number of sates in a trellis and the computational effort of the branch metrics. There exist other receivers based on oversampling [7] or LD, among which only those based on LD can achieve simultaneously simplified front-end and state reduction in the detector. In the case of single user systems, it is observed that the LD-based receiver and the proposed receiver have the same performance in terms of BER and complexity as was stated in [12]. If those most significant components are used in LD, a simpler trellis can be constructed with negligible performance loss in single user systems. However, this does not work in multiuser systems, where a degradation up to 1.5 dB (around BER 10^−3^) is observed [9].

Actually, LD is rather a decomposition of the phase trajectories than the CPM signal itself. Therefore, LD-based front ends for CPM multiuser systems must consider the signals of individuals [9]. This implies a linearly increased number of filters in LD receivers. To conclude, we say that the LD-based receiver and proposed receiver are roughly comparable in single user systems [12] but may differ when proceeding to multiuser systems.

As to the proposed receiver, there are three ways to simplify the complexity by (1) reducing the number of effective filters *N*_*E*_, (2) reducing the size of *s*_*R*_(*t*, **a**) by letting *L*_*R*_ < *L*, or combing (1) and (2) together. These methods are evaluated numerically in the next section.

## 4. Numerical Results

The performance of the proposed receivers is evaluated in different scenarios. Different multiuser schemes are designed to demonstrate the impact on the performance by different modulation parameters such as Δ*f*, *θ*, *L*_*R*_, and the number of effective filters being used *N*_*E*_′.

In Table 1, for *h* = 0.5, two systems based on a raised-cosine frequency response of duration 2 (i.e., binary CPM2RC) or a rectangular shape of duration 2 (i.e., binary CPM2REC) are detected by MSK-based receivers. Different systems are evaluated according to the measurements in Section 3. It is evident that the proposed receiver can reduce the complexity significantly while the average energy loss is marginal with much less required matched filters. The number of filters required by the LD-based receiver is *K* · 2^*P*(*L*−1)^(2^*P*^ − 1) [12], where *P* is defined as 2^*P*−1^ < *M* ≤ 2^*P*^. To make a fair comparison, we should take into account that the filters of LD are usually real valued whose durations are several symbol intervals, while the filters of proposed receivers are complex valued and their duration is one symbol interval. Since the proposed receivers are designed to process the superimposed signals, the signals of new users do not always increase the dimensions due to the strong correlation between CPM signals. This is also observed in Table 1, where *N*_*E*_ increases slowly or even remains the same while increasing *K*.

**Figure 2:**
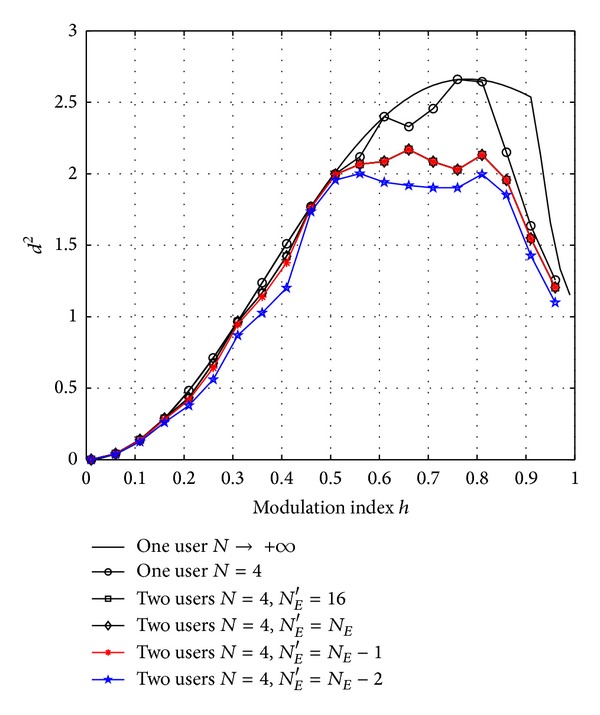
The minimum achievable distance *d* ^2^ versus the modulation index *h*, optimum (*L*_*R*_ = *L*) receivers.

The optimum (*L*_*R*_ = *L*) receivers for different two-user binary CPM2RC systems are considered in Figure 2. In these systems, the main concern is to examine the impact of *N*_*E*_. The conventional front-end consisting of *M* ^*KL*^ = 16 filters is also shown as a reference. To reduce the complexity further, some (i.e., *N*_*E*_′) most significant dimensions are employed. For a given observation length *N*, the minimum achievable distance *d* ^2^ versus modulation index *h* is shown. It is observed that *N*_*E*_ effective filters are sufficient to reconstruct the signals and no degradation is made. When *N*_*E*_′ = *N*_*E*_ − 1, a marginal but negligible degradation is observed. When *N*_*E*_′ = *N*_*E*_ − 2, the gap is up to 0.6 dB (*h* ∈ [0,0.5]). Therefore, *N*_*E*_′ = *N*_*E*_ − 1 would be a good choice. It is also observed that CPM-based multiuser systems also suffer from weak index [1]. It should be pointed out that Δ*f* = 0, which makes this multiuser system the most band-efficient scheme. The parameter Δ*ϕ* is optimized to maximize *d* ^2^. These designed multiuser systems can approach the single user systems asymptotically with no sacrifice of bandwidth efficiency. The use of Δ*ϕ* is justified.

**Figure 3:**
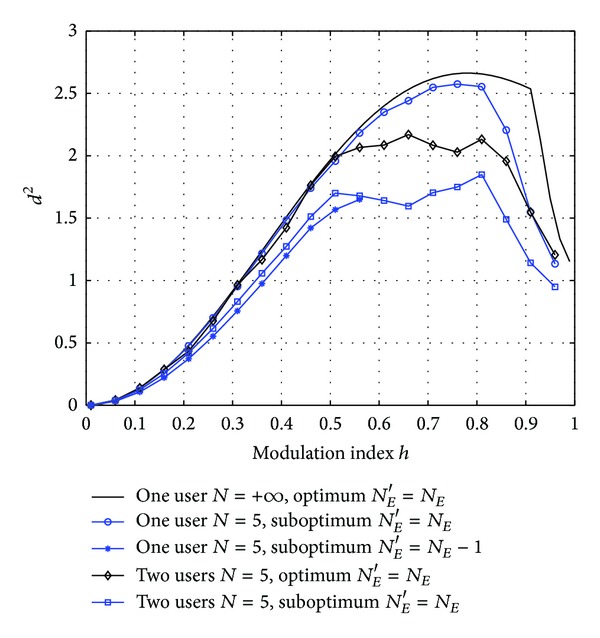
The minimum achievable distance *d* ^2^ versus the modulation index *h* for suboptimum (*L*_*R*_ < *L*) receivers. Δ*f* = 0 and Δ*ϕ* is optimized.

The proposed suboptimum receivers based on 1REC are considered in Figure 3 with optimized Δ*ϕ* and Δ*f* = 0. This frequency response was particularly suited for single user binary 2RC systems [14]. The performances of the suboptimum and the optimum receivers are compared. It is seen that these suboptimum receivers have a performance loss no more than 1 dB. However, due to the severe degradation caused by the dimension reduction observed in single user systems, this technique is not adopted in these multiuser receivers.

**Figure 4:**
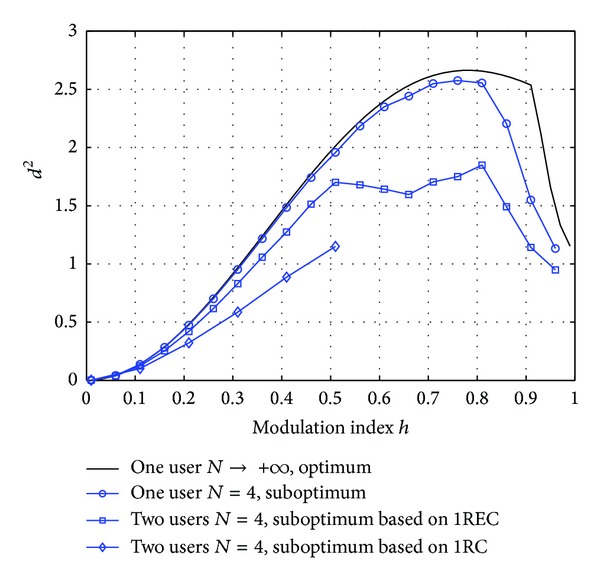
The minimum achievable distance *d* ^2^ versus the modulation index *h*. Δ*f* = 0 and Δ*ϕ* is optimized.

Presented in Figure 4 is a comparison of two suboptimum receivers based on 1REC and 1RC, respectively. It is seen that the 1RC based receiver is 1.5 dB worse than the 1REC based receiver. This figure implies that the frequency response particularly suited for single user system is probably a better choice than shortening the frequency response of the transmitter directly.

Based on the results and discussion above, it can be seen that the proposed receivers are successfully implemented in CPM-based systems. The designed multiuser systems with optimized parameters almost have an identical BER as the corresponding single user systems. It can be expected that the performance can be further improved using the method presented in [15]. Another issue is the choice of different parameters, especially Δ*ϕ* and Δ*f*. In our case, there is no need to use Δ*f* > 0. However, for different systems the conclusions may differ. It is also noticed that, for some modulation indices such as *h* ∈ [0.5,0.8], a severe degradation is observed. This is due to the fact that a longer observation length (i.e., *N*) is required. Anyhow, it is evident that a properly designed CPM-based system has an asymptotically identical BER with the corresponding single user systems.

## 5. Conclusion

A class of simplified maximum-likelihood receivers is proposed for CPM-based multiuser systems. The basic idea is to perform detection over a low-dimensional signal space such that the computational effort is reduced significantly (even exponentially in some cases). The performance of the proposed receiver is evaluated by means of analysis and justified by the minimum achievable Euclidean distance. The impact of modulation parameters is examined in detail for the designed schemes, which reveal that the proposed receiver requires less filters than some existing schemes and can be further reduced with negligible performance loss. Though the main concern is designing maximum-likelihood receivers, it should be obvious that the presented principles can be generalized to other suboptimum receivers (such as [17]) with few modifications.

## Figures and Tables

**Table 1:** Comparison and performance analysis of some MSK-based receivers.

Transmitter	*K*	Δ*f*	Number of effective filters *N*_*E*_		LD front end	Energy loss	Complexity reduction
			{*s*(*t*, **a**)}	{*s*_*R*_(*t*, **a**)}			
Binary CPM 2REC	1	0.00	3	2	2	0.010	2-fold
	2	0.00	3	2	4	0.015	4-fold
		0.25	4	3		0.086	4-fold
		0.5	4	3		0.100	4-fold
		0.00	3	2	10	0.021	32-fold
	5	0.25	6	5		0.098	32-fold
		0.5	8	6		0.026	32-fold

Binary CPM 2RC	1	0.00	3	2	2	0.002	2-fold
	2	0.00	4	2	4	0.002	4-fold
		0.25	5	3		0.058	4-fold
		0.5	5	3		0.098	4-fold
	5	0.00	4	2	10	0.003	32-fold
		0.25	8	5		0.101	32-fold
		0.5	9	6		0.031	32-fold
